# Different impacts of parental psychological and behavioral control on the cultivation of advantageous strength in Chinese rural adolescent

**DOI:** 10.3389/fpsyg.2024.1349386

**Published:** 2024-08-30

**Authors:** Rong Wang, Min Liu, Yinghong Sun, Qian Li

**Affiliations:** ^1^School of Education and Psychology, University of Jinan, Jinan, China; ^2^Sichuan Vocational College of Chemical Technology, Luzhou, China

**Keywords:** parental control, personal growth initiative, meaning in life, future planning, adolescent

## Abstract

**Introduction:**

Numerous studies have focused on the mental and behavioral problems or negative emotions of adolescents when examining the impact of parental control. However, limited research has explored the relationship between parental control and adolescents’ future planning, neglecting the distinctions between parental psychological and behavioral control, as well as the significant roles of personal growth initiative and meaning in life. The present study aims to investigate the differential effects of parental psychological control and behavioral control on the future planning of rural adolescents in China.

**Methods:**

A sample of 909 individuals (13.60±0.93 years old, 470 boys and 439 girls) completed a self-report questionnaire anonymously. The study utilized the Adolescent Future Orientation Questionnaire, Parental Control Questionnaire, Adolescents’ Personal Growth Initiative Scale, and Meaning in Life Questionnaire.

**Results:**

The findings revealed that parental control significantly influenced the future planning of Chinese rural adolescents, with psychological control and behavioral control exerting distinct impacts in this process. Specifically, parental psychological control was found to have a direct negative effect on adolescents’ future planning, while also exhibiting a positive influence due to the masking effect of meaning in life; it did not negatively affect adolescents’ future planning through their personal growth initiative. On the other hand, parental behavioral control was observed to directly and positively impact adolescents’ future planning, as well as positively influence it through the mediating role of personal growth initiative and the chain mediating role of personal growth initiative and meaning in life.

**Discussion:**

These results suggest that the influence of parental control on adolescents’ future planning is not a simple, singular mechanism, but rather a multi-layered and complex process, yielding mixed outcomes as a result of psychological control, behavioral control, and other factors. This complexity should be taken into consideration in educational practices and future research endeavors.

## 1 Introduction

Future planning refers to the process of individuals setting goals and formulating and implementing plans; it includes two main dimensions: exploration and investment (Nurmi, 1991). Adolescence is a period of contemplating the future and preparing for the transition to adulthood. It is also a crucial period for the rapid development, differentiation, and expansion of future orientation (Nurmi, 1993). During this time, contemplating and making decisions about the future are vital for adult life. Empirical research indicates that thinking about the future can help adolescents discover more of their potential and envision a positive self, which can ultimately bring them hope (Shipp and Aeon, 2019). Therefore, it is essential to understand the current state of future planning among adolescents, explore its influencing factors, and implement corresponding measures to enhance adolescents’ future planning ability.

Bronfenbrenner’s (1986) ecological systems theory points out that the influence of the family on adolescents’ development is the most direct and enduring. There is a strong correlation between family parenting styles and adolescents’ future plans (Neblett and Cortina, 2006; Xu et al., 2019; Zhang and Zhang, 2021). As a key family parenting style, parental control has an important impact on adolescents’ future planning. It is initially extracted from the parenting style (Baldwin, 1948) and becomes more acceptable to be distinguished as psychological control and behavioral control, which are usually seen as a negative parenting style and a positive parenting style, respectively, (Steinberg, 1990; Barber, 2002). Behavioral control includes parents controlling adolescents’ behavior through active questioning, restrictive discipline, and other strategies, as well as regulating their behavior; while psychological control is when parents use strategies such as inducing feelings of guilt in their children, withdrawal of love, and authoritative interference to constrain children’s thoughts and emotions (Barber, 2002; Shek and Law, 2014). Empirical research has also found that, in general, psychological and domineering parental control have a negative impact on adolescents’ development, while parental behavioral control has a positive impact on their development (e.g., Chen et al., 2000; Inguglia et al., 2022). Parental behavioral control does not have a negative impact on children’s future development and can even reduce some of their children’s deviant behavior problems (Liu and Guo, 2010). Parents’ strict supervision over adolescents’ behavior can positively impact their future planning. In contrast to behavioral control, high-level psychological control tends to result in children with poor mental health or development (Liu et al., 2020; Lin et al., 2020; Cristiano et al., 2020; Yu et al., 2021; Pérez et al., 2021; Geng et al., 2022; Sun and Ban, 2022; Li and Gu, 2023; Bi et al., 2024; Liu et al., 2024; Zhan et al., 2024; Țepordei et al., 2024; Yang, 2024; Yu et al., 2024). Excessive use of psychological control by parents can easily make children dependent, thereby hindering the formation of their self-identity, which is not conducive to positive future planning for teenagers (Yu, 2021) and negatively predicts their future orientation (Li and Gu, 2023). The present study speculates that parental control can impact adolescents’ future planning, and that behavioral control and psychological control play different roles in this process.

In addition to family factors, individual factors also play an important role in adolescents’ future planning and development. An example is personal growth initiative, which refers to the tendency of individuals to consciously and proactively work on self-improvement and self-actualization (Robitschek, 1998). Existing research has pointed out that personal growth initiative could affect the setting of individual goals and the strategies and means to achieve these goals (Luyckx and Robitschek, 2014; Xu et al., 2019). Individuals with stronger personal growth initiative have a clearer vision and well-defined plans about their future life, have stronger motivation to explore themselves and the environment, and are more able to calmly face issues associated with career development (Robitschek and Cook, 1999). Autonomy is the most significant feature of personal growth initiative (Ahmad et al., 2013). Deci and Ryan’s (1985) self-determination theory holds that individuals have a tendency to actively grow and self-regulate. This basic, evolving autonomy need and internal motivational tendency can guide individuals to proactively explore external information (Koestner, 2008). The process of adolescents’ future planning is a process of determining their own development goals and using strategies to achieve them, which is a kind of self-determination behavior. Personal growth initiative embodies both initiative (autonomy, internal motivation, associated with self-determination theory) and promotion (development, future pointing, associated with future planning). Meanwhile, personal growth initiative is influenced by family factors (Xu et al., 2019; Tao et al., 2024). Parental psychological control may be detrimental to the acquisition of adolescent autonomy, while behavioral control may contribute to the development of adolescent autonomy (McElhaney et al., 2009). The present study speculates that adolescents’ personal growth initiative may have an impact on their future planning under the influence of parental control.

Meaning in life refers to an individual’s exploration, perception, and experience of the meaning, purpose, and value of life, including two dimensions: having a sense of meaning and seeking a sense of meaning (Steger et al., 2006). Theoretical research shows that one of the key functions of meaning in life is the temporal integration of the past, present, and future, encouraging individuals to focus on future possibilities (Baumeister et al., 2013). The motivational aspect of meaning in life suggests that it can influence individuals’ attitudes towards the future (Miao and Gan, 2019; Miao et al., 2021), encourage them to consider future goals, and guide them in achieving these goals (Hooker et al., 2018). Empirical studies have also shown that meaning in life can enhance individuals’ ability to cope adaptively with the future (Sedikides and Wildschut, 2018). Recognizing that the life is meaningful motivates people to expand their goals from the present to the future, leading them to focus more on the future (Miao and Gan, 2019; Miao et al., 2021). Obtaining meaning in life is inseparable from the influence of family factors. Researches show that adolescents’ meaning in life is associated with parental supervision (Malinakova et al., 2019) and authoritative parenting (Roman et al., 2015). Parental supervision can assist adolescents developing behaviors that align with societal expectations. Parental behavioral control has a positive predictive effect on adolescents’ sense of meaning in life, while psychological control has a negative predictive effect (Shek et al., 2021). Psychological control can lead to emotional instability and insecure attachment issues in adolescents, hindering them from forming meaningful life goals (Romm et al., 2020). The present study hypothesizes that meaning in life may influence adolescents’ future planning under the influence of parental control.

In addition, there is a significant correlation between personal growth initiative and meaning in life (Ghaderi et al., 2019). Promoting personal growth initiative can lead individuals to perceive a sense of meaning in life (Borowa et al., 2020). Developing meaning in life may be a challenging process, requiring continuous efforts to achieve personal growth and to respond positively to adversity. Individuals who frequently utilize personal growth initiative skills to bring about positive changes may be more likely to perceive meaning in life and consequently experience greater satisfaction (Borowa et al., 2020). Enhancing the personal growth initiative of high school students can improve their sense of meaning in life and effectively reduce the occurrence of psychological problems (Wang and Han, 2020). Therefore, it is reasonable to believe that enhancing personal growth initiative can nurture an individual’s sense of meaning in life, with a focus on creating life meaning through personal growth (Steger et al., 2006). This correlation is closely linked to the impact of family factors. For example, parental psychological control infringes upon the independence and autonomy of adolescents, leading to behavior driven by external forces rather than their own values and interests, thereby impeding their exploration of meaning in life (Soenens and Vansteenkiste, 2010).

Based on previous research, the present study hypothesizes that personal growth initiative and meaning in life may play a chain mediating role in the influence of parental control on adolescents’ future planning. The mechanism of action may vary based on psychological control and behavioral control. Existing studies, particularly those on psychological control, have primarily focused on adolescents’ mental and behavioral problems or negative emotions (Liu et al., 2020; Lin et al., 2020; Cristiano et al., 2020; Yu et al., 2021; Pérez et al., 2021; Geng et al., 2022; Sun and Ban, 2022; Li and Gu, 2023; Bi et al., 2024; Liu et al., 2024; Zhan et al., 2024; Țepordei et al., 2024; Yang, 2024; Yu et al., 2024), with limited attention to future planning and personal growth initiative. With the emergence of positive psychology, researchers have started focusing on the strengths of children and adolescents, emphasizing the exploration and cultivation of positive factors in their development. Aligned with the goals of positive psychology to enhance human potential and facilitate future success, it is imperative to investigate adolescents’ personal growth initiative, meaning in life, future planning, and the impact of parental control. Furthermore, the underlying mechanisms regarding the relationships between different forms of parental control and adolescents’ future planning and development remain inadequately explored. Research on Chinese junior high school students’ future planning is limited. This study concentrates on junior high school students to test the hypothesis due to its practical significance in contemporary China. The recent reform in the college entrance examination system has introduced a diversified admission process and comprehensive evaluation criteria, granting students more autonomy in selecting academic paths. Junior high school students will encounter a crucial decision (choosing between ordinary high school or vocational high school) after the entrance examination. The Guidelines for Mental Health Education (revised in 2012) by the Ministry of Education of the People’s Republic of China emphasize the importance of addressing junior high school students’ future planning, a topic that will be explored in this study.

## 2 Materials and methods

### 2.1 Participants and measures

Using a convenience sampling method, 1,000 questionnaires were distributed to students from grade 1 to grade 3 in a public junior high school in Anyang city, Henan Province. The response rate of the questionnaires was 90.9%. The final valid sample included 909 adolescents (13.60 ± 0.93 years old; 470 males, 51.71%; 439 females, 48.29%). All tests were conducted in Mandarin Chinese. Additionally, a demographic questionnaire compiled by the researchers was employed to collect information on the participants’ demographic characteristics, such as gender, age, grade, parents’ marital status, parents’ educational level, and daily guardian. Distribution of valid participants on demographic variables is presented in Table 1.

**Table 1:** Distribution of valid participants on demographic variables (*N* = 909).

Demographic variable	Category	Count	Percentage (%)
Gender	Male	470	51.71
	Female	439	48.29
Grade	Grade 1	376	41.40
	Grade 2	335	36.90
	Grade 3	198	21.80
Parents’ marital status	In Marriage	865	95.20
	Divorced	44	4.80
Father’s educational level	Junior High School or Below	697	76.70
	Technical Secondary School or High School	194	21.30
	College Degree or Above	18	2.00
Mother’s educational level	Junior High School or Below	700	77.00
	Technical Secondary School or High School	186	20.50
	College Degree or Above	23	2.50
Daily guardian	Parents	472	51.90
	Father Only	56	6.20
	Mother Only	208	22.90
	Other Relatives	173	19.00

### 2.2 Questionnaires

#### 2.2.1 Parental control

The Chinese version of the Parental Control Questionnaire prepared by Wang et al. (2007) was adopted in this study. It comprised two sub-questionnaires: Parental Psychological Control and Parental Behavioral Control. The Parental Psychological Control Questionnaire contained 18 questions and mainly measured parents’ behavior across three dimensions: causing guilt, withdrawal of love, and dictating power. The Parental Behavioral Control Questionnaire contained 16 questions and mainly measured parents’ behavior across two dimensions: active inquiry and restraint. A five-point scale was used, ranging from “1 = completely inconsistent” to “5 = completely consistent.” The higher the score was, the higher the parental control was. In this study, the Cronbach’s *α* coefficient of the Parental Psychological Control Questionnaire was 0.86, and the Cronbach’s *α* coefficient of the Parental Behavioral Control Questionnaire was 0.86. The internal consistency was good.

#### 2.2.2 Personal growth initiative

The Adolescents’ Personal Growth Initiative Scale (APGIS) compiled by Guo and Ye (2016) was adopted. The questionnaire comprised 24 questions across five dimensions: growth internal cause, growth self-determination, growth autonomy, growth seeking, and growth external cause. A six-point scale was used, ranging from “1 = very inconsistent” to “6 = very consistent.” The higher the score, the higher the personal growth initiative. In this study, the Cronbach’s *α* coefficient of this questionnaire was 0.93, indicating good internal consistency.

#### 2.2.3 Meaning in life

The Meaning in Life Questionnaire (MLQ) compiled by Steger et al. (2006) and revised by Wang (2013) was used. The questionnaire comprised two subscales: the Presence of Meaning (MLQ-P) and the Search for Meaning (MLQ-S). Each had five questions, totaling ten questions. The second question was reverse-scored. A seven-point scoring system was employed, with scores ranging from 1 to 7. The higher the score, the stronger the meaning in life. In this study, the Cronbach’s *α* coefficient of this questionnaire was 0.81, indicating good internal consistency.

#### 2.2.4 Future planning

The Future Planning of Education and Occupation sub-questionnaires in the Adolescent Future Orientation Questionnaire compiled by Nurmi et al. (1996) and revised by Zhang et al. (2006) were used. Each sub-questionnaire comprised two dimensions: exploration and investment, with a total of 14 items. A five-point rating was used, ranging from 1 to 5 points. The higher the score was, the more exploration and investment were in the future. In this study, the Cronbach’s α coefficient of the total Future Planning Questionnaire was 0.85; the Cronbach’s *α* coefficient of the Future Education Planning sub-questionnaire was 0.73; and the Cronbach’s *α* coefficient of the Future Career Planning sub-questionnaire was 0.79. The internal consistency of the questionnaire was good.

### 2.3 Procedure

The present study was approved by the Research Ethics Committee of the University of Jinan. After obtaining consent from the students and their parents, the school’s full-time psychology teacher assisted in distributing the questionnaire during a psychology class. The researchers explained the purpose of the research to the students and emphasized the principles of voluntary participation, anonymity, and truthful answers. Subsequently, the class was utilized as a group for group testing. The questionnaires required approximately 45 min to complete and were promptly collected afterward.

### 2.4 Statistical analysis

We used SPSS Version 25.0 along with the PROCESS v3.5 application developed by Hayes (2018) for statistical analysis. The PROCESS v3.5 application enabled us to accurately estimate the effect sizes and confidence intervals of each pathway, providing a robust statistical foundation for the reliability and validity of our research findings. Using PROCESS, we analyzed the impact of parental psychological control, with behavioral control as a covariate, and the impact of behavioral control, with psychological control as a covariate.

The specific analytical steps included: firstly, we conducted descriptive analysis and Pearson correlation analysis using SPSS v25.0 to understand the basic characteristics of the data and explore the relationships between variables; then, we used Model 6 of PROCESS v3.5 for chain mediating effect analysis, obtaining 95% bias-corrected confidence intervals through 5,000 bootstrap resampling iterations to test the significance of indirect effects. Indirect effects were considered significant if the 95% confidence interval did not include zero (Erceg-Hurn and Mirosevich, 2008). Through these analyses, we were able to gain a deeper understanding of the complex mechanisms among parental control, personal growth initiative, meaning in life, and future planning.

## 3 Results

### 3.1 Correlation analysis of major study variables

Table 2 presented the means, standard deviations, and Pearson correlation coefficients for parental behavioral control, parental psychological control, personal growth initiative, meaning in life, and future planning. The correlation analysis showed that adolescents’ parental behavioral control was positively correlated with parental psychological control (*r* = 0.26, *p* < 0.001), personal growth initiative (*r* = 0.43, *p* < 0.001), meaning in life (*r* = 0.36, *p* < 0.001), and future planning (*r* = 0.26, *p* < 0.001); parental psychological control was significantly positively correlated with meaning in life (*r* = 0.10, *p* < 0.01), but it was not significantly correlated with personal growth initiative (*r* = 0.06, *p* > 0.05) and future planning (*r* = −0.06, *p* > 0.05); personal growth initiative was significantly positively correlated with meaning in life (*r* = 0.70, *p* < 0.001) and future planning (*r* = 0.47, *p* < 0.001); meaning in life was significantly positively correlated with future planning (*r* = 0.47, *p* < 0.001). The results of the correlation analysis provided preliminary evidence for the hypotheses.

**Table 2:** Means, standard deviations, and Pearsons correlation coefficients of all variables.

Variables	M ± SD	1	2	3	4	5	6
1. Gender	0.52 ± 0.50	–					
2. Age	13.60 ± 0.93	0.04	–				
3. Behavioral control	3.36 ± 0.74	0.03	0.01	–			
4. Psychological control	2.74 ± 0.71	0.15^***^	0.08^*^	0.26^***^	–		
5. Personal growth initiative	4.10 ± 0.89	0.05	−0.05	0.43^***^	0.06	–	
6. Meaning in life	4.70 ± 1.06	0.04	−0.07^*^	0.36^***^	0.10^**^	0.70^***^	–
7. Future planning	3.06 ± 0.61	−0.01	0.02	0.26^***^	−0.06	0.47^***^	0.47^***^

Gender: male = 1, female = 0.

### 3.2 Test of the mediating effects of personal growth initiative and meaning in life between parental psychological control and future planning

From the perspective of general mediation, although the correlation between parental psychological control and adolescents’ future planning was not significant (*p* > 0.05), existing studies have shown that parental psychological control, personal growth initiative, meaning in life, and future planning were related to each other. There is a close relationship between them, so the existence of a masking effect can be considered (Wen and Ye, 2014).

After controlling for participants’ grade and gender which showed significant differences for the study variables (Table 3), and controlling for parental behavioral control as a covariate, the results (Table 4) showed that parental psychological control could positively impact on junior high school students’ meaning in life (*β* = 0.07, *p* < 0.05) and negatively impact their personal growth initiative (*β* = −0.08, *p* < 0.05) and future planning (*β* = −0.11, *p* < 0.001). The personal growth initiative had a positive impact on meaning in life (*β* = 0.80, *p* < 0.001) and future planning (*β* = 0.16, *p* < 0.001); the meaning in life of junior high school students had a positive impact on their future planning (*β* = 0.17, *p* < 0.001).

**Table 3:** Grade and gender difference test on the research variables (*N* = 909).

Research variables	Demographic variables	Group	M	SD	t/F	Group comparison
Future planning	Grade	①grade 1	3.03	0.61	5.65^**^	② > ③
		②grade 2	3.14	0.64		
		③grade 3	2.97	0.56		
Parental psychological control	Gender	①male	2.84	0.67	4.45^***^	① > ②
		②female	2.63	0.74		
Parental behavioral control	Grade	①grade 1	3.34	0.74	5.37^**^	② > ①, ③
		②grade 2	3.45	0.72		
		③grade 3	3.24	0.74		
Personal growth initiative	Grade	①grade 1	4.13	0.93	3.97^*^	①, ② > ③
		②grade 2	4.16	0.84		
		③grade 3	3.95	0.88		
Meaning in life	Grade	①grade 1	4.78	1.06	4.11^*^	①, ② > ③
		②grade 2	4.71	1.04		
		③grade 3	4.52	1.07		

*p < 0.05, **p < 0.01, ***p < 0.001, the same below.

**Table 4:** Regression model of the effect of parental psychological control on adolescents’ future planning.

Variables	β	t	p	*R* ^2^	F
Step1 outcome variable: personal growth initiative					
Control variable 1 Gender	0.09	1.62	>0.05	0.20	55.34
Control variable 2 Age	−0.05	−1.80	>0.05		
Control variable 3 Parental behavioral control	0.55	14.57	<0.001		
Predictor Parental psychological control	−0.08	−2.04	<0.05		
Step2 outcome variable: meaning in life					
Control variable 1 Gender	0.01	0.10	>0.05	0.50	182.12
Control variable 2 Age	−0.05	−1.83	>0.05		
Control variable 3 Parental behavioral control	0.08	2.12	<0.05		
Predictor Parental psychological control	0.07	2.03	<0.05		
Mediator 1 Personal growth initiative	0.80	25.69	<0.001		
Step3 outcome variable: future planning					
Control variable 1 Gender	−0.02	−0.49	>0.05	0.30	58.27
Control variable 2 Age	0.04	2.09	<0.05		
Control variable 3 Parental behavioral control	0.17	7.14	<0.001		
Predictor Parental psychological control	−0.11	−4.30	<0.001		
Mediator 1 Personal growth initiative	0.16	5.68	<0.001		
Mediator 2 Meaning in life	0.17	7.41	<0.001		

The *β*-value refers to the unstandardized coefficient.

**Figure 1:**
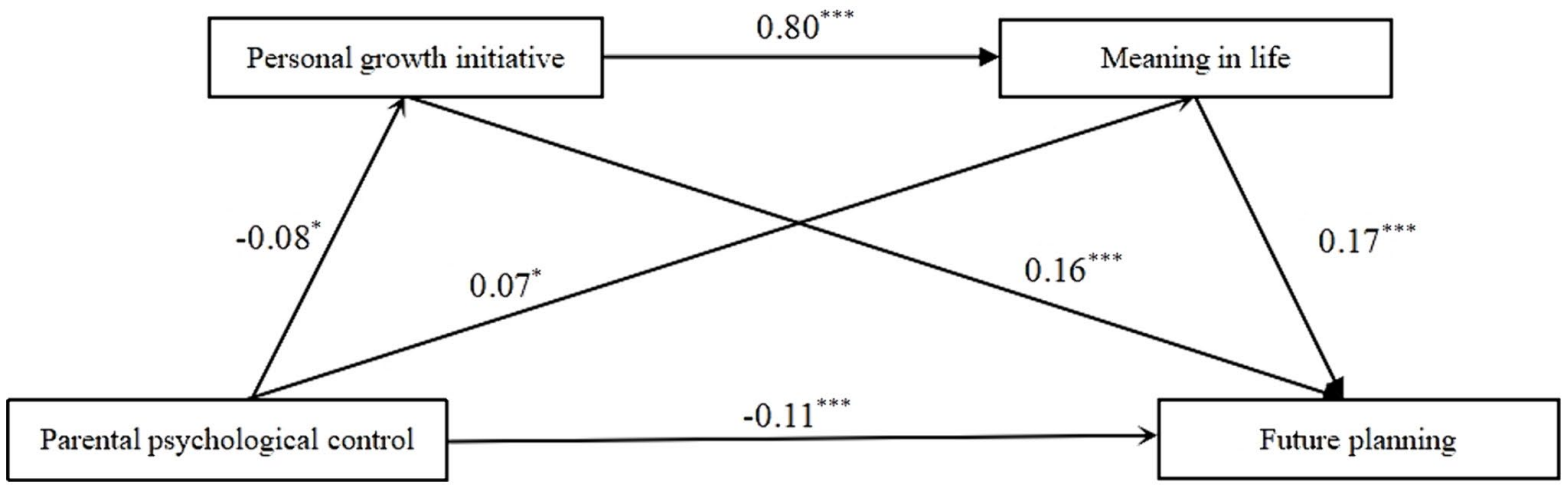
The mediating effect model of personal growth initiative and meaning in life on the relationship between parental psychological control and future planning.

The results of the mediating effect test were shown in Table 5, and the path diagram was shown in Figure 1. The results found that the 95% confidence intervals of the total effect, direct effect, and path 2 of the mediating model did not include 0, and the 95% confidence interval of the total indirect effect included 0, indicating that the total effect and direct effect were significant, the total indirect effect was not significant, but the effect of parental psychological control → meaning in life → future planning was significant.

**Table 5:** Multiple mediating effect analysis of parental psychological control on adolescents’ future planning.

	Effect	Boot SE	Bootstrap 95% CI		Relative effect ratio (%)
			Low	High	
Total effect	−0.12	0.03	−0.18	−0.06	100%
Direct effect	−0.11	0.03	−0.16	−0.06	91.7%
Total indirect effect	−0.01	0.01	−0.04	0.02	–
Path 1: Parental psychological control → Personal growth initiative → Future planning	−0.01	0.01	−0.03	0.01	–
Path 2: Parental psychological control → Meaning in life → Future planning	0.01	0.01	0.001	0.03	8.3%
Path 3: Parental psychological control → Personal growth initiative → Meaning in life → Future planning	−0.01	0.01	−0.02	0.001	–

The effect ratio is the ratio of the effect to the total effect.

### 3.3 Test of the mediating effects of personal growth initiative and meaning in life between parental behavioral control and future planning

After controlling for participants’ grade and gender, which showed significant differences for the study variables (Table 3), and controlling for parental psychological control as a covariate, the results showed (Table 6) parental behavioral control could positively impact on junior high school students’ personal growth initiative (*β* = 0.55, *p* < 0.001), meaning in life (*β* = 0.08, *p* < 0.05) and future planning (*β* = 0.06, *p* < 0.05); junior high school students’ personal growth initiative positively impacted their meaning in life (*β* = 0.80, *p* < 0.001) and future planning (*β* = 0.16, *p* < 0.001); and junior high school students’ meaning in life positively impacted their future planning (*β* = 0.17, *p* < 0.001).

**Table 6:** Regression model of the effect of parental behavioral control on adolescents’ future planning.

Variables	β	t	p	*R* ^2^	F
Step1 outcome variable: personal growth initiative					
Control variable 1 Gender	0.09	1.62	>0.05	0.20	55.34
Control variable 2 Age	−0.05	−1.80	>0.05		
Control variable 3 Parental psychological control	−0.08	−2.04	<0.05		
Predictor Parental behavioral control	0.55	14.57	<0.001		
Step2 outcome variable: meaning in life					
Control variable 1 Gender	0.01	0.10	>0.05	0.50	182.12
Control variable 2 Age	−0.05	−1.83	>0.05		
Control variable 3 Parental psychological control	0.07	2.03	<0.05		
Predictor Parental behavioral control	0.08	2.12	<0.05		
Mediator 1 Personal growth initiative	0.80	25.69	<0.001		
Step3 outcome variable: future planning					
Control variable 1 Gender	−0.02	−0.49	>0.05	0.30	58.27
Control variable 2 Age	0.04	2.09	<0.05		
Control variable 3 Parental psychological control	−0.11	−4.30	<0.001		
Predictor Parental behavioral control	0.06	2.38	<0.05		
Mediator 1 Personal growth initiative	0.16	5.68	<0.001		
Mediator 2 Meaning in life	0.17	7.41	<0.001		

The *β*-value refers to the unstandardized coefficient.

**Figure 2:**
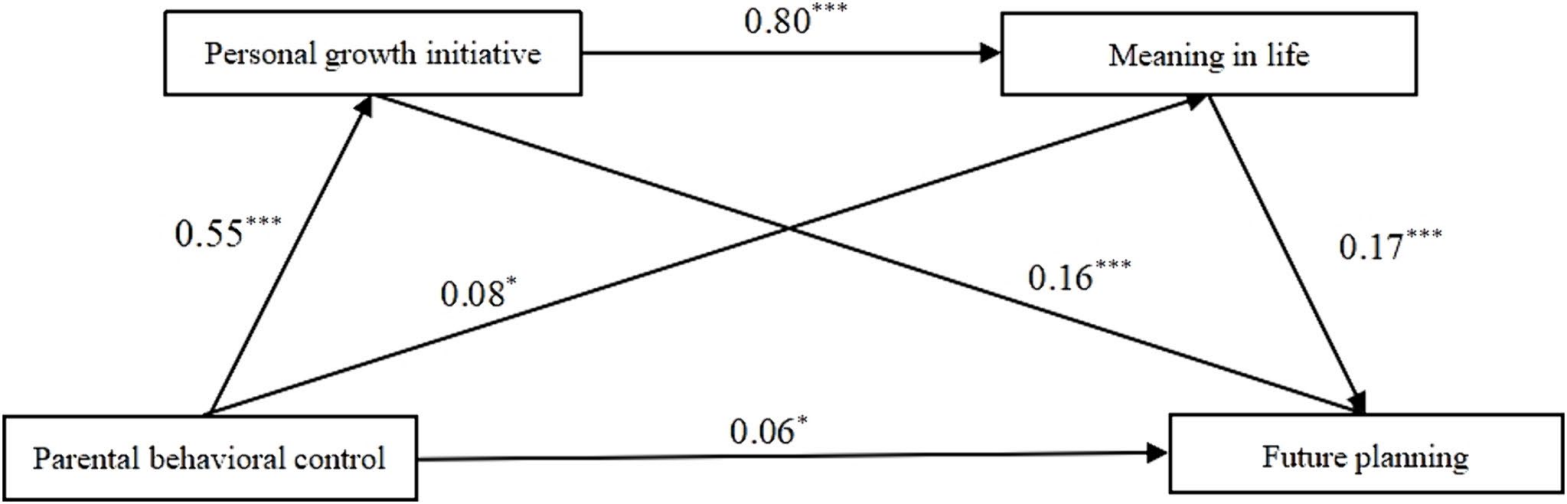
The mediating effect model of personal growth initiative and meaning in life on the relationship between parental behavioral control and future planning.

The results of the mediating effect test were shown in Table 7, and the path diagram was shown in Figure 2. The results found that the 95% confidence intervals of the total effect, direct effect, and total indirect effect of the mediating model did not include 0, indicating the significance of both direct and indirect effects in this mediating model. The indirect effect of parental behavioral control on adolescents’ future planning occurred through two paths: Path 1, which included parental behavioral control → personal growth initiative → future planning; Path 3, which included parental behavioral control → personal growth initiative → meaning in life → future planning. The 95% confidence intervals of the two paths did not include 0, indicating that the mediating effect of personal growth initiative was significant, and the chain mediating effect produced by personal growth initiative and meaning in life was significant. However, the effect of path 2 parental behavioral control → meaning in life → future planning, was found to be insignificant.

**Table 7:** Multiple mediating effect analysis of parental behavioral control on adolescents’ future planning.

	Effect	Boot SE	Bootstrap 95% CI		Effect ratio (%)
			Low	High	
Total effect	0.24	0.03	0.19	0.30	100%
Direct effect	0.06	0.03	0.01	0.12	25%
Total indirect effect	0.18	0.02	0.14	0.21	75%
Path 1: Parental behavioral control → Personal growth initiative → Future planning	0.09	0.02	0.05	0.12	37.5%
Path 2: Parental behavioral control → Meaning in life → Future planning	0.01	0.01	−0.001	0.03	–
Path 3: Parental behavioral control → Personal growth initiative → Meaning in life → Future planning	0.07	0.01	0.05	0.10	29.2%

The effect ratio is the ratio of the effect to the total effect.

## 4 Discussion

The study confirmed the hypothesis that parental control impacted adolescents’ future planning, with personal growth initiative and meaning in life playing a role in this process. The effects varied depending on whether the type of parental control was psychological control or behavioral control.

### 4.1 The influence of parental psychological control on adolescents’ future planning: the direct effect, indirect effect, and inconformity

Consistent with previous research findings indicating that parental psychological control has a negative impact on adolescents’ development, the direct effect of parental psychological control on adolescents’ future planning was significant and negative in the present study. The total indirect effect was insignificant, but it’s worth noting that the path of psychological control → meaning in life → future planning was significant and positive. This finding contradicted the direct effect in the present study and also contradicted the previous results (e.g., Romm et al., 2020; Shek et al., 2021).

The consensus among most researchers is that parental psychological control is a negative parenting style that reduces adolescents’ sense of control over their lives and negatively predicts adolescents’ sense of meaning in life (Romm et al., 2020; Shek et al., 2021). In this study, parental psychological control promoted adolescents’ meaning in life, and thus positively promoted adolescents’ future planning. The result is similar to several studies in China which find that parental psychological control has no significant negative impact on junior middle school students’ creative self-efficacy (Liu et al., 2020), and no significant negative impact on junior middle school students’ autonomous motivation (Ren et al., 2017). There were four possible reasons. First, there might be other mediating or moderating variables between parental psychological control and adolescents’ meaning in life. For example, self-differentiation can play a partial mediating role between adolescents’ emotional neglect and meaning in life (He et al., 2023a). According to the maintenance and flow model of meaning in life, when the lack of one kind of psychological resource leads to a reduction in an individual’s meaning in life, the individual will improve their sense of meaning by looking for other psychological resources (Zhao et al., 2017). Parental psychological control might stimulate other psychological resources for adolescents. When teenagers encounter difficulties in getting along with their parents, they will have a strong desire to get support from teachers and peers, thereby promoting their connection with school and improving their sense of meaning. Second, there were cultural differences in parenting styles. Studies have shown that in collectivist cultures such as Chinese culture, the negative influence of parental psychological control may be weak or even non-existent (Cheung and Pomerantz, 2011). In Chinese culture, more teenagers are willing to accept an authoritarian parenting style. Compared to Western teenagers, Chinese teenagers tend to interpret parental control as a sign of care and love (Soenens and Vansteenkiste, 2010; Liu et al., 2020). With a high degree of parental psychological control, the adolescents might be more likely to feel a close family connection and interdependence, achieve emotional integration, and thus feel that life was meaningful. Another empirical cross-cultural study also supports these findings (Chou and Chou, 2020). Cultural differences between the U. S. and Taiwan in the structures and levels of parental psychological and behavioral control, and the association between the two controls are found among emerging adults (Chou and Chou, 2020). The researchers discussed that the results from the U. S. sample suggested the conceptual distinction between behavioral and psychological control, reflecting the different functions of psychological control and behavioral control, while the results from the Taiwanese sample showed a positive association between behavioral control and psychological control and were in concordance with most findings from the Chinese mainland. Third, this study did not distinguish the different dimensions of psychological control which might have different influences on Chinese adolescents. For example, love withdrawal has a negative effect on adolescents’ psychological well-being, while guilt induction does not (Fang et al., 2022). Love withdrawal directly and negatively predicts adolescents’ academic achievement, while guilt induction and authority assertion are not significant direct predictors (Xu et al., 2024). The cultural difference between China and America also varies by these dimensions (Chou and Chou, 2020). Chinese parents may be more likely to use authority assertion and less likely to use love withdrawal (Liu et al., 2020). Fourthly, it was related to different life stages. The present study included junior high school students. The impact of parental psychological control on adolescents’ sense of meaning in life may show different patterns in early and late adolescence (Shek et al., 2021). Parental psychological control itself changes as time passes (He et al., 2023a,b; Sun et al., 2017). The current research findings on the influence of parental psychological control on adolescents’ sense of meaning in life are limited and inconclusive, and therefore need to be further investigated and verified. Whether directly or indirectly enhancing the sense of meaning in life will have a positive effect on individuals (Liu and Gan, 2010). Having a sense of meaning in life can help individuals integrate temporal continuity and motivate them to focus on their future potential (Baumeister et al., 2013; Miao and Gan, 2019; Miao et al., 2021), and promote them to consider future goals and guide them to achieve these goals (Hooker et al., 2018).

Personal growth initiative positively impacted future planning. However, unlike the meaning in life it did not have a mediating effect on the relationship between parental psychological control and adolescents’ future planning. This finding was specific to the sample in the present study and was also linked to the acceptance of parental psychological control in China. Personal growth initiative encompasses both initiative (autonomy, internal motivation) and promotion (development, future orientation). In Chinese culture, particularly in rural areas, parents prioritize their children’s future, even if their parenting styles were not always scientifically sound, democratic, or appropriate. Consequently, parental psychological control might only hinder Chinese rural adolescents’ autonomy within personal growth initiative, without necessarily impeding development or future orientation within this initiative. As a result, the impact of psychological control on personal growth initiative was not significant enough for personal growth initiative to act as a mediator between psychological control and future planning. Moreover, there were distinctions in the roles of fathers and mothers in Chinese rural families, which could affect the different aspects of their children’s personal growth initiative. The multidimensional nature of personal growth initiative should also be taken into account in future research.

In summary, although parental psychological control had a negative effect on adolescents’ future planning in terms of a direct relationship, its impact was alternatively positive due to the intervention of meaning in life. Meaning in life played a masking role, inhibiting the relationship between the predictor and outcome variables (Wen and Ye, 2014). In other words, the influence of parental psychological control on adolescents’ future planning might be both positive and negative, and its positive effect could be realized through increasing adolescents’ meaning in life. It could not negatively impact adolescents’ future planning through their personal growth initiative. Due to the complexity of psychological control and adolescents’ psychological development, further research is needed.

### 4.2 The influence of parental behavioral control on adolescents’ future planning: the direct effect and indirect effect

In line with previous research findings, parental behavioral control, identified as a form of positive parenting behavior (Wang et al., 2007), played a positive role in adolescents’ future planning in this study. This was observed through the direct effect, the mediating effect of personal growth initiative, and the chain mediating effect of personal growth initiative and meaning in life, which represents an advancement in existing research.

Parental behavioral control promoted adolescents’ future planning by influencing their personal growth initiative. Personal growth initiative refers to the proactive actions individuals take to achieve personal development, which can ignite their motivation to explore themselves and their surroundings. A higher level of personal growth initiative enables individuals to handle career development tasks more effectively (Robitschek and Cook, 1999). Positive parenting styles are strong predictors of adolescents’ personal growth initiative development (Hirata and Kamakura, 2018). Parental guidance and monitoring help adolescents identify their weaknesses and areas for improvement. Adolescents who recognize their own shortcomings and have a clear direction for progress demonstrate a high level of personal growth initiative, enabling them to generate more ideas and plans for their future.

The mediating effect of meaning in life on the relationship between behavioral control and future planning was insignificant, but the chain mediating effect was significant. This was also related to the sample in the present study. Chinese culture places much more emphasis on affective ties in family relationships. Behavioral control includes more rational instruction with relatively less emotional involvement. Therefore, it did not significantly influence future planning through meaning in life which was related to affections. However, rational behavioral control could impact personal growth initiative, which in turn could affect meaning in life. Promoting individuals’ personal growth initiative can improve their sense of meaning in life (Borowa et al., 2020; Vaksalla and Hashimah, 2015; Wang and Han, 2020). A high level of meaning in life can effectively promote adolescents’ future planning (Baumeister et al., 2013; Hooker et al., 2018; Miao and Gan, 2019; Miao et al., 2021). Of course, fathers’ and mothers’ different roles need to be considered in future research as well.

Generally, both psychological control and behavioral control impacted the future planning of Chinese rural adolescents, but their mechanisms were different. Psychological control could directly and negatively impact adolescents’ future planning, but it could positively influence their future planning through the mediating effect of meaning in life. It could not negatively impact their future planning through adolescents’ personal growth initiative. Behavioral control could directly and positively influence adolescents’ future planning, and could also positively influence it through the mediating effect of personal growth initiative and the chain mediating effect of personal growth initiative and meaning in life. In essence, different types of parental control impacted adolescents’ future planning through distinct pathways and psychological mechanisms. The role of meaning in life was particularly noteworthy. These findings might be attributed to the fact that the participants were junior high school students in rural China. Their understanding and acceptance of parental control might be unique, warranting further research.

These findings imply that parents should strive to become qualified parents by providing their children with proper guidance and paying attention to their own psychological characteristics. To better plan for adolescents’ future education, it is essential to effectively utilize the connection between parental control and adolescents’ psychological factors in order to have a positive impact.

## 5 Limitations

It is important to note that this conclusion is limited to the cross-sectional study conducted with a specific group of junior high school students within the context of Chinese culture, treating psychological control as unidimensional instead of multidimensional. Therefore, any extensions of these findings should be approached with caution. Future research would benefit from longitudinal studies or comparative studies among different age groups. It is necessary to deeply examine the potential and different functions of the psychological control dimensions. Additionally, beyond personal growth initiative and meaning in life, a wider range of factors and psychological contents should be incorporated or studied. For example, self-identity, self-differentiation, big five personality, parental career-related behavior, teachers, peers, and school activities may affect adolescents’ future planning. According to the ecological systems theory (Bronfenbrenner, 1986) and existing research on meaning in life and future planning (e.g., He et al., 2023a,b; Zhang and Zhang, 2021), they may be important environmental or individual factors affecting adolescents’ future planning. Cross-cultural studies should also be conducted to gain a better understanding of the comprehensive mechanism of parental control on adolescents’ future planning.

## 6 Conclusion

The findings of this study indicate that the influence mechanism of parental control on adolescents’ future planning is not a singular, simple mechanism. Instead, it is multi-layered and complex, with mixed outcomes varying according to psychological control, behavioral control, and other factors. This should be considered comprehensively in educational practice and future research.

## Data Availability

The original contributions presented in the study are included in the article/supplementary material, further inquiries can be directed to the corresponding author.
